# Employing Common Morality to Address Ethical Issues in Genetics Research With Indigenous People: The *Ọmọlúàbí* Ideology

**DOI:** 10.1002/ajpa.70246

**Published:** 2026-04-19

**Authors:** Iyunoluwa J. Ademola‐Popoola, Laura S. Weyrich

**Affiliations:** ^1^ Department of Anthropology Pennsylvania State University University Park Pennsylvania USA; ^2^ Department of Bioethics Pennsylvania State University University Park Pennsylvania USA; ^3^ Rock Ethics Institute, Pennsylvania State University University Park Pennsylvania USA; ^4^ One Health Microbiome Center, Huck Life Sciences Institute University Park Pennsylvania USA; ^5^ Australian Centre for Ancient DNA, School of Biological Sciences and The Environment Institute, The University of Adelaide Adelaide South Australia Australia

**Keywords:** culture‐specific common morality, genetics research ethics, indigenous groups, Ọmọlúàbí

## Abstract

Genetics research has transformed our understanding of human diversity, providing insights into human evolution, migration, and health. Despite its contributions, many ethical challenges remain unresolved, particularly in studies involving Indigenous or non‐Western populations. Existing ethical frameworks rooted in Principlism—autonomy, beneficence, non‐maleficence, and justice—often fail to address issues like data ownership, informed consent, and community engagement. Furthermore, frameworks designed to address some of these limitations, such as the FAIR and CARE principles, can emphasize autonomy and universal principles over local norms that are specific to a group's cultural and communal values and diversity. We propose integrating cultural‐specific common moralities alongside existing frameworks for genetics research with Indigenous peoples. As an example, we highlight the Ọmọlúàbí ideology of the Yoruba people, which offers a group‐centered approach rooted in respect, humility, integrity, and communal responsibility. Ọmọlúàbí emphasizes co‐creation and collaboration between researchers and communities, ensuring research aligns with local moral landscapes and community priorities. By recognizing Indigenous cultural and moral perspectives, researchers can use Ọmọlúàbí to foster trust, inclusivity, and ethical rigor, moving beyond a one‐size‐fits‐all model. This approach can guide all stages of genetics research—from project development and data collection to interpretation and dissemination—embedding local cultural values alongside global guidelines. It also underscores building relationships through participant observation and respecting community‐specific traditions and authority structures. Adopting a culturally sensitive framework like Ọmọlúàbí offers a path toward genetics research that is both respectful and equitable, bridging the divide between scientific progress and the preservation of Indigenous identities.

## 1 Introduction

In recent decades, genetics research has been at the vanguard of scientific advances, continually expanding the frontier of what is known about human diversity—historically and at present. Research in this area has significantly contributed to anthropology, biology, medicine, and society (Gibbon et al. 2018). In anthropology, human genetics research has undeniably shaped our understanding of human origins (Fu et al. 2014; Vernot et al. 2016; Wolf and Akey 2018), trajectories of human migration (Choudhury et al. 2020), past and present health (Ruth et al. 2020; Zhang et al. 2020), and the complexities of human diversity (Anastassopoulou et al. 2020; Langmyhr et al. 2021). It has also driven methodological and technological advancements and critical reevaluations of race, identity, and ethics in scientific research (Lasisi and Shriver 2018; Quillen et al. 2019).

Despite these advances, genetics research consistently produces complex ethical dilemmas and empirical epistemological concerns (Fletcher 2023; McGuire et al. 2008). Thus, genetics scholars worked to develop an ethical consciousness surrounding research praxis (Boddington 2012; Kowal et al. 2023; Wagner 2019; Wagner et al. 2020). Specifically, issues of data management, data ownership, autonomy, and informed consent, among others, have repeatedly arisen (Godard et al. 2003; Handsley‐Davis et al. 2023; Horton and Lucassen 2023; Rego et al. 2020). However, resolutions to these issues are often outpaced by the expansion and scale of new genetics studies. For example, genetics research often inadequately considers the rights and needs of community groups—especially Indigenous populations (Kowal 2015).

At the forefront of many of these issues lies an ethical ideology that was developed following the *Belmont Report* (United States. National Commission for the Protection of Human Subjects of Biomedical 1978)—Principlism. Principlism posits autonomy, beneficence, non‐maleficence, and justice. The framework holds that these prima facie principles are equally important; however, in actual practice, a significant focus is placed on autonomy, arguing that competent individuals have the right to make decisions regarding themselves and their data (Beauchamp and Childress 1994). This conventional Western approach regards research participants as individual, autonomous agents capable of providing informed and voluntary consent (Beauchamp 2010; Beauchamp and Childress 1994).

However, these principles are found to be inadequate for appropriately protecting Indigenous communities. First, the emphasis on individual autonomy directly contrasts with the relational and collective ethical systems that structure many Indigenous communities, where personhood, rights, and responsibilities are negotiated relationally through kinship, lineage, community authority, or other means (Dvorakova 2019). Consent, therefore, is not merely an individual act but a socially embedded process. When Principlism assumes autonomy is solely individual, it obscures the collective forms of decision‐making that many Indigenous groups view as ethically essential. Emphasis on individual autonomy obscures Indigenous societies' communal cultural lifeways and value systems, ignoring non‐Western cultural variations (Beauchamp 2008; Knoppers and Chadwick 2005; Tsosie et al. 2021). Further, these principles fall short in genetics research on unidentified or ancient people, where decisions about genetic sequencing of an individual(s) may be placed in the hands of a community and not the individual's genetic descendants (Fitzpatrick et al. 2016; Kowal et al. 2023; Tsosie et al. 2021). The notion of individual consent may not fully capture the ethical concerns of communities for whom genetic data can have deep cultural, spiritual, legal, and social implications, neglecting the potential for harm that can arise from misrepresenting or misusing genetic information, which may affect entire communities (Fitzpatrick et al. 2016; Harding et al. 2012). For instance, decisions about using ancestral remains or genetic material in many Indigenous societies are inherently collective, often involving elders, spiritual leaders, and entire communities (Walker 2008). Furthermore, Western ethical standards prioritize short‐term procedural consent over long‐term relational accountability, neglecting researchers' enduring responsibility toward the communities in which they engage. These gaps can result in ethical breaches, even when researchers believe they adhere to best practices, perpetuating distrust and marginalization (Nadeau et al. 2022; Simonds and Christopher 2013).

These divergent orientations shape how beneficence and non‐maleficence are interpreted. Because beneficence is always culturally defined, what researchers view as beneficial may differ substantially from community understandings of well‐being or long‐term impact. If beneficence is defined primarily through researcher or institutional priorities, these definitions may fail to capture community‐level concerns, thereby undermining both beneficence and non‐maleficence. The principle of justice also requires deeper examination. Justice in Principlism is often interpreted procedurally (e.g., fair subject selection, equitable distribution of burdens and benefits), but these procedural forms are insufficient for communities who continue to experience structural violence, historical dispossession, and systemic exclusion from the very systems that govern research (Ashworth et al. 2021; Beauchamp 2008; Pratt et al. 2020). Western justice frameworks rarely address historical harms, epistemic injustices, or violations of Indigenous sovereignty, issues that deeply shape how Indigenous communities experience research. Without confronting these structural and historical realities, justice remains shallow and cannot meaningfully address the inequities Indigenous peoples continue to face (Beauchamp 2008; Claw et al. 2018; Garrison 2013; Pratt et al. 2020). These shortcomings are not simply theoretical but have practical consequences for how research is conducted, interpreted, and experienced by the communities whose lives and histories it affects.

Given the limitations of Principlism, researchers developed additional specific principle‐based frameworks to address individualism and guide the ethical conduct of genetics research. For example, approaches using findability, accessibility, interoperability, and reusability (FAIR) are the most common in the management of data, while applying a collective benefit, authority to control, responsibility, and ethics (CARE) scaffold can more broadly guide the research process (Carroll et al. 2021). The FAIR principles were developed to address emerging ethical issues identified in the reusability of data among other research teams. A focus on principles targeted toward data management was developed primarily due to the demands by funding bodies and government agencies for researchers across multiple fields to provide information on their data management plans (Wilkinson et al. 2016). FAIR principles focus on the technical aspects of data management and sharing to promote data usability and interoperability in the scientific community (Carroll et al. 2020; Wilkinson et al. 2016). While this framework aims to promote growth in the scientific community, one of the significant constraints of the FAIR principles is their weak emphasis on the societal context, data autonomy, and ethical implications of data reuse, especially for community‐based research (Carroll et al. 2021, 2020). For example, they do not consider the effects of sharing data on underrepresented communities or marginalized groups, which are often the sources of highly valuable data but hardly ever benefit from the insights or output derived from that data (Reardon and TallBear 2012). Moreover, there are no guidelines for sharing data between different research groups or labs, especially if human participants are involved in research. Such an omission increases the risk of a power imbalance, in which the better‐positioned research entities can be expected to benefit more from shared data and, in effect, will be relatively unfair to smaller or under‐financed groups (Carroll et al. 2020). This also places decision‐making in the hands of the researchers and can diminish the role of participants or communities in this process.

As a result, the CARE framework emerged as a principles‐oriented framework to reinforce the rights of Indigenous people to control data regarding their people, land, cultures, knowledge, and relationships with their territories and resources (Carroll et al. 2021; Martinez Cobo 1982). The CARE Principles provide an incredibly valuable foundation for ethical research. Although they are global in origin and application, they have become most widely referenced and operationalized in North American contexts. However, successful implementation of CARE requires deep cultural grounding within a localized context. For example, the “E‐Ethics” component of the CARE principles is only relevant when a researcher has a deep knowledge of local cultural, social, or historical realities. Otherwise, researchers may enact the ethics component ineffectively or harmfully, particularly in cross‐cultural or international genetics research, because a researcher's ethical realities may not align with a community's reality. Importantly, CARE does not treat local strategies as optional additions; rather, it explicitly requires that high‐level principles be operationalized through locally designed Indigenous data sovereignty and data governance models (Carroll et al. 2021, 2020). Recognizing that without engagement with community‐specific values, priorities, and lived experiences, the implementation of keystone CARE principles could become superficial or generalized, ultimately reducing the depth and effectiveness of these important ethical research practices. To address this gap, culturally grounded moral frameworks are needed to guide the interpretation and enactment of CARE's high‐level ethical commitments within specific community contexts. Despite these commitments, current genetics research practices continue to fall short, as geneticists have worked to adopt the above principles, yet fail to represent Indigenous groups' full panoply of interests (Carroll et al. 2021).

Here, we argue that interpreting local morality in conjunction with existing national and international guidelines and existing frameworks is one such solution to these issues. It builds on prior ethical recommendations by proposing a more nuanced and culturally sensitive approach to research ethics related to genetics. Specifically, we argue that there is no universal common morality. Thus, research ethics cannot be viewed as a globally applicable praxis; rather, appropriate research ethics must be locally specific in ways that reflect the worldviews of the group in question. This approach recognizes that while principle‐based ethical frameworks, such as CARE, FAIR, and Principlism, offer a valuable approach to conducting ethical genetic research, their universal adaptability is problematic due to cultural and regional variations in community norms (Kowal et al. 2023; Oyinloye 2021). However, they can be enhanced by being used in conjunction with local values.

This approach is situated within the plurality of cultures, which celebrates the diversity of cultures within a society, emphasizing the importance of respecting and valuing cultural differences while promoting mutual understanding and coexistence among diverse groups (Kiser 1949). It asserts that each cultural group within a larger society has the right to maintain its distinct identity, practices, and beliefs (Arasaratnam 2013; Kiser 1949). This ideology recognizes the richness and vibrancy that cultural diversity brings to society, enriching the social fabric and contributing to greater social cohesion. By embracing cultural diversity, societies can foster greater inclusivity, respect, and appreciation for individual and collective differences, ultimately contributing to a more equitable and harmonious coexistence. In many instances, what Indigenous groups consider ethical is in stark contrast with the standards and approaches enforced by Western institutions (Tilley 2000). These approaches obscure the inherent plurality of cultures. Cultural pluralism holds that smaller groups within a larger group maintain their unique identities, emphasizing the relativity of individual cultures (Kiser 1949).

From this conceptual standpoint, this paper uses a culture‐specific, common morality framework unique to individual groups to direct future genetics research (Clouser and Gert 2004). To exemplify this approach, this work examines the morality framework of the Yoruba, a group of people from Southwestern Nigeria with shared ideas and cultural practices (Ogundiran 2020). The Yoruba are one of the largest ethnic groups in West Africa, with cultural groups located in various regions, including Benin, Togo, Brazil, Saint Dominican Republic, and Jamaica (Akande et al. 2022). Among the Yoruba, the *Ọmọlúàbí* philosophy is paramount (Akindele et al. 2022). “*Ọmọlúàbí́”* is a Yoruba ideology that conveys the ideal person, emphasizing qualities of good character, integrity, moral uprightness, and responsibility.

In the following sections, we provide a model for ethical genetics research among Indigenous groups. To do this, we examine how the Yoruba^1^ people have incorporated their shared morality into communal and social organizations, and how this can inform conducting genetics research with cultures that share Yoruba morality. This strategy recognizes the value of honoring cultural viewpoints and adapting ethical frameworks to embrace community values and beliefs for more inclusive and culturally aware genetics research praxis. To ensure that the advantages of genetic developments are available to and respectful of diverse societies, this paper seeks to contribute to the establishment of more culturally sensitive approaches to genetics research ethics. Incorporating these community‐specific moral standards into existing global and national guidelines enriches the ethical framework, ensuring a more comprehensive approach that reduces harm and risks for communities that want to participate in genetics research.

## 2 Culture‐Specific Common Morality

Culture‐specific common morality can be defined as the collection of ethical standards that individuals within a particular society or social group consciously or subconsciously adopt when confronted with moral dilemmas (Clouser and Gert 2004). It explains that there is a shared moral compass firmly ingrained in cultural groups (DeGrazia 2003). Such culture‐specific common morality embodies the community's shared sense of “right” and “wrong”; therefore, it governs how community members are expected to behave and interact. These morals are pivotal in establishing a society's normative foundation, creating a benchmark against which its members' actions are evaluated (Gert 2004). Common morality is an ideal that becomes deeply embedded in the subconscious of individuals socialized into specific cultural contexts (Clouser and Gert 2004). Thus, common morality underlies individuals' moral decisions and behaviors.

Common morality is multifaceted and has been the subject of extensive philosophical and sociological inquiry (Beauchamp 2003). It is essential to explore the intricate ways these ethical approaches to morality evolve and manifest within the specific dynamics of a given society. This includes examining transmission across generations and adaptation to change. Furthermore, examining common morality can illuminate an understanding of individual and collective decision‐making processes and how a society's moral fabric is formed (Neog 2007). Subconscious assimilation of an ethical perspective can profoundly affect an individual's moral outlook and conduct by influencing perceptions of right and wrong, a sense of duty and responsibility toward others, and a willingness to adhere to social norms (Beauchamp 2003). The existence and persistence of common morality within different societies can underscore communal ethical practice and foster social cohesion (Alaei et al. 2023).

As a result, common morality frameworks allow for in‐depth analysis of how shared norms play out in specific cultural contexts. Therefore, this paper explores the *Ọmọlúàbí́* of the Yoruba from Southwestern Nigeria to show how a culture's shared morality is a valuable ethical heuristic for conducting genetics research. In what follows, this paper argues that an *Ọmọlúàbí́* approach offers a widely adopted ideology embedded in common morality that is capable of being adopted by genetics researchers working with humans. This paper demonstrates that respectfully integrating and applying communal morality frameworks, such as *Ọmọlúàbí́*, can help mitigate the culturally conscious ethical inadequacies and gaps inherent in Principlism and the FAIR and CARE principles in the conduct of genetics research with communal groups.

## 3 *Ọmọlúàbí* ^2^ as a Model of Culture‐Specific Common Morality

There is a growing consensus within the research community that advocates for a shift in the roles of researchers and participants, with participants playing a more active and substantial role in the research process, moving beyond the traditional role of mere subjects (Douglass 2022; Oyinloye 2021, 2022). This paradigm shift views researchers and participants as co‐creators in the research process, fostering a more equitable and collaborative relationship (Bader et al. 2023; Douglass 2022; Kowal et al. 2023). Building upon these recent calls for a more inclusive and participatory research model (Bader et al. 2023; Douglass 2022), the *Ọmọlúàbí́* epistemological framework emerges as a culturally tailored approach for researchers seeking to responsibly and ethically interact with research participants as genuine co‐creators (Oyinloye 2021). The crux of this perspective lies in recognizing that the principles of *Ọmọlúàbí́* extend beyond mere ethical considerations. They also serve as a framework for engaging with participants in a manner that aligns with the concept of co‐creation (Oyinloye 2021). In essence, *Ọmọlúàbí́* encourages genetics researchers to conduct their research with a deep respect for the participants' cultural values and moral principles and actively involve participants in shaping the research process (Oyinloye 2022). This entails collaborative decision‐making, mutual understanding, and shared responsibility for the research outcomes. By embracing *Ọmọlúàbí́* as a guardrail for conducting genetics research, researchers can transcend conventional, hierarchical dynamics that have historically characterized researcher‐participant relationships (Oyinloye 2021; Råheim et al. 2016).

Furthermore, this approach fosters a sense of ownership and empowerment among research participants, who are no longer passive subjects but active contributors to the research endeavor (Douglass 2022). This shift toward co‐creation is particularly relevant when working with communities like the Yoruba in Southwest Nigeria, where cultural sensitivity and community engagement are paramount (Ferguson et al. 2015; Minoi et al. 2019; Oyinloye 2021). For instance, there is a popular saying among the Yoruba, “*enikan kii je awade”*, which translates as no individual has the name ‘everybody’. This proverb foregrounds the interrelationship among members of society by insisting on collective responsibility and the common good. It promotes the idea that no single person can embody or achieve everything independently, fostering a communal society where collaboration, mutual support, and collective decision‐making are highly valued. This cultural principle underscores the Yoruba worldview, which holds that individual success is intrinsically tied to the prosperity and harmony of the larger community. As such, *Ọmọlúàbí* provides researchers with a guide to navigating an intricate cultural terrain in a manner that demonstrates respect for the unique cultural identity of the Yoruba people.

For example, the Yoruba people revere “*Ọmọlúàbí́”* both as an ideal moral person and as a broader ideology that embodies the ideals of honor, hard work, respect for others' rights, and commitment to community well‐being through deeds and actions (Akindele et al. 2022; Olanipekun 2017). Central to being viewed by the community as an *Ọmọlúàbí́* is personal integrity (Akindele et al. 2022; Olanipekun 2017). Here, integrity incorporates principles of *ìwà* (character). Among the Yoruba, the moral principles of *ìwà* have different variants, including *ìwà ìtẹríba* (respect), *òtítọ́* (truth), *isẹ́ sis*e (hard work), *ìwà ìrẹ̀lẹ̀* (humility), *ìronú* (critical thinking), *ọ̀rọ̀ sísọ̀* (culturally grounded speech), *mímọ̀ rírí àwùjọ* (giving back to the community), and *Ajọṣe* (communalism), among others (Oyinloye 2021). These various principles of *ìwà* transcend individual consciousness to become a communally based common morality (Oyeshile 2007). Members of the Yoruba community are brought up from birth to exhibit characteristics of *Ọmọlúàbí́*, and thus will have to show *ìwà* throughout their lives even when physically absent from the community (Oyeshile 2007). As such, *Ọmọlúàbí́* can be understood as the shared moral compass of the Yoruba people, guiding their individual and communal ethical decision‐making and shaping day‐to‐day existence.

For researchers seeking to conduct genetics research with Yoruba communities, embracing the qualities of *Ọmọlúàbí* is necessary to earn trust. Failure to adhere to this ideology could lead to unfavorable reactions from individuals and the community, including hostility and refusal to engage with the researcher. *Ọmọlúàbí* requires new community entrants, such as researchers, to engage respectfully (*ìwà ìtẹríba*), reciprocate by giving back to society (*mímọ̀ rírí àwùjọ*), and conduct themselves with humility (*ìwà ìrẹ̀lẹ̀*). Many Indigenous groups across the globe expect similar conduct from researchers (Seehawer 2018). Thus, following the common morality framework within respective communities is a valuable and ethical praxis for genetic researchers.

For instance, researchers must comprehend and respect the community's social structure when collecting human samples from living or ancestral individuals. Beyond the community structure, understanding how the culture perceives the human body and its constituent parts, including social beliefs and settings, is critical (Makinde 1984). Appreciating these perspectives is fundamental to both sample collection and laboratory research processes. Unlike Western cultures, which often prioritize individuality (Cohen et al. 2016), the Yoruba culture emphasizes communalism (Oyeshile 2007), with specific individuals assuming leadership roles within the community. Prior approval must be sought from the community. Moreover, before research can commence, rituals may need to be performed to appease local deities. Therefore, conducting genetics research requires that the community members collectively make decisions about sample collection and data use, and rituals be made if necessary (Oyinloye 2021). This collective decision‐making process contrasts sharply with the individualized approach pervasive in the West (Cohen et al. 2016; Oyeshile 2007). Thus, genetics research must comply with the given cultural contexts.

This compliance requires researchers to develop their methodologies with these communities and respect the authority structures in the community. This may involve engaging in dialog and negotiations with community leaders, obtaining community consensus, and adhering to culturally specific protocols. In Western research contexts, particularly those governed by institutional guidelines, the primary requirement for conducting research among human populations typically involves obtaining approval from institutional review boards (IRBs) and discussion with individual participants (Grady 2015; Israel 2014). However, this does not necessarily align with the cultural and communal dynamics of Yoruba and other Indigenous communities. Researchers must recognize that a “one‐size‐fits‐all” approach regarding ethical and cultural considerations associated with genetics research involving different populations is inadequate. Thus, the *Ọmọlúàbí* ideology offers an example of adopting a culturally sensitive approach to ethical genetics research. This approach can be applied to genetics research conducted in collaboration with Indigenous populations where researchers engage the community to understand the culture‐specific common morality to develop a similar ethical framework.

## 4 *Ọmọlúàbí́:* A Culture‐Specific Common Morality in Practice

**FIGURE 1:**
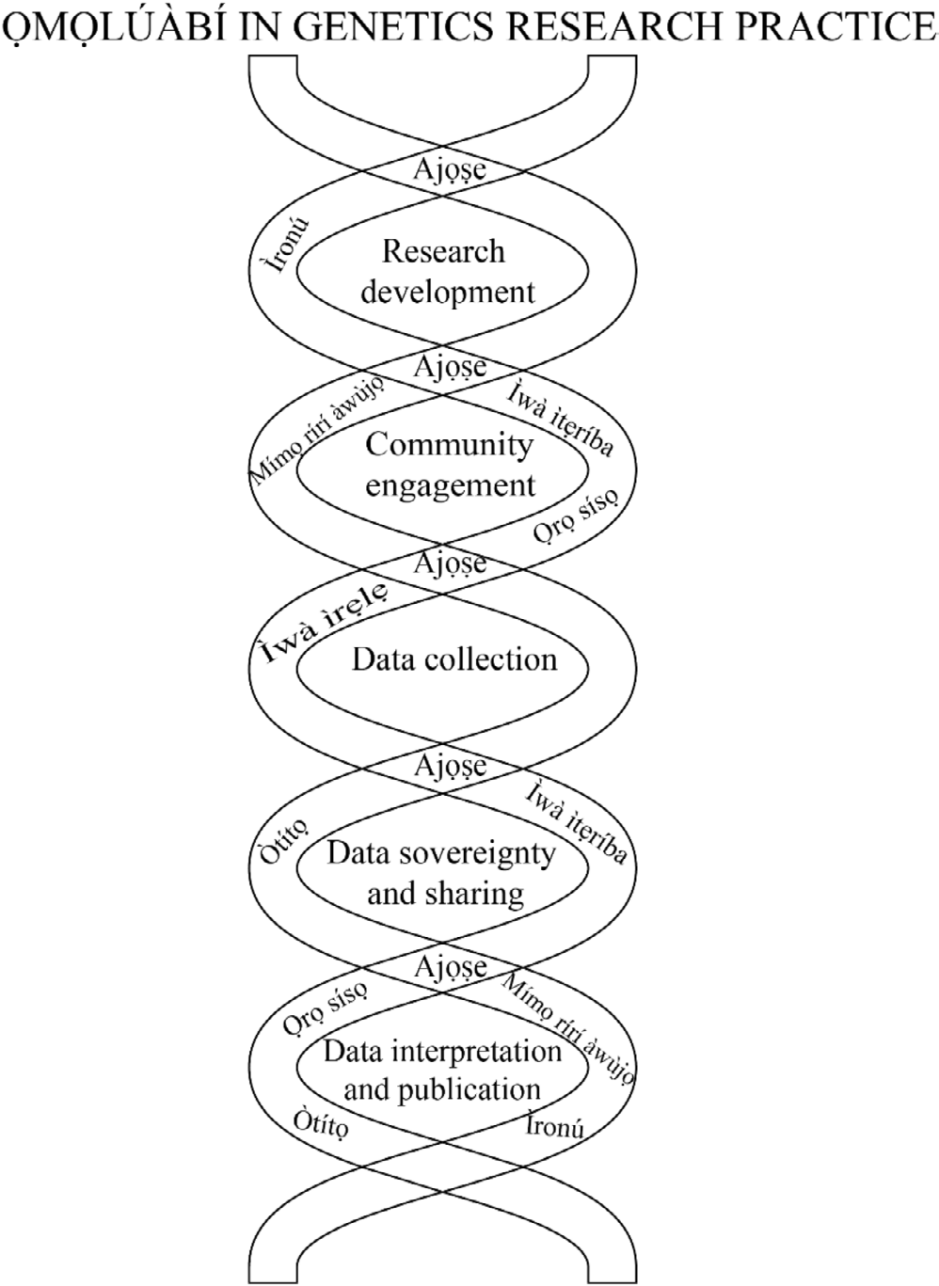
This visualization uses the form of the DNA double helix which is reminiscent of the Guilloché motif carved into Yoruba Opon Ifá (divination trays) to illustrate the intertwined nature of research stages and the Omoluabi tenets. In Yoruba epistemology, Ifá, the deity of wisdom and knowledge, speaks through diviners who use binary logic to derive guidance through odu. Similarly, this figure conveys the paired, dynamic relationship between core Yoruba ethical values and the stages of the research process. The values do not act in isolation but are woven into each stage, with Ajọṣe (community collaboration) serving as a central strand linking all stages together.

Oyinloye (2022) posits a compelling practical argument for incorporating the *Ọmọlúàbí́* principle within research (particularly during data collection and community engagement) by underscoring the importance of how participants perceive researchers and how such perceptions influence the success—or failure—of research endeavors. First, this paper argues that the *Ọmọlúàbí́* ideology extends beyond the data collection and initial community engagement phase. Second, this paper argues that it extends to any genetics research, including research on human remains or cultural artifacts. As a result, it is crucial to recognize the ethical underpinnings of this moral ideology as it extends into laboratory settings. The ways in which genetic materials are handled during research and the terminologies employed to refer to individuals included in research should also be governed by the moral norms of the culture, as guided by *Ọmọlúàbí́* among the Yoruba people. Researchers must identify and adopt culturally appropriate ethical strategies that harmonize their scientific pursuits with the moral values of the population participating in the study. These two critical dimensions must not exist in isolation; instead, they should operate interdependently, facilitating a symbiotic relationship where insights from one dimension inform and enrich the other, thereby fostering a holistic and culturally sensitive approach to research ethics (Figure 1).

Ọmọlúàbí́, being a social etiquette, guides one to become a felicitous member of society. Thus, lacking this etiquette would negatively impact the research process and the researcher's interaction with society. In fact, starting with the project‐development phase, it is pertinent to explore and fully understand the moral principles of the participating population (Figure 1). The development of research questions must show that a researcher has *ìronú* (critical thinking) that reflects *Ọmọlúàbí́*. In developing genetics‐related questions, the researcher must demonstrate that they have given deep thought (*ìronú*) to the questions they ask, which must also fit into the contextual and moral epistemologies of the Yoruba people. The researcher must also have a sense of community (*ajọṣe*), ensuring that the research project will not harm community members or the community as a whole.

Conceptualization of the research should also be conducted in collaboration with community members to ensure it is an endeavor the community is interested in or that benefits them. Beyond the conceptualization phase, continued community engagement during the data‐collection and manuscript‐writing phases is pertinent. With the Yoruba, for example, core dimensions of *Ọmọlúàbí* all play essential roles in ensuring that community engagement is fruitful and that the community members are willing to provide samples. Researchers must involve the people as a collective using the tenets of *ajọṣe* (communalism). *Ajọṣe* prescribes collective engagements until a resolution is made. Conducting genetics research among the Yoruba, researchers must show *ìwà ìtẹríba* (respect) to host communities and pay homage to the remains of elders/ancestors. Human remains must be viewed as the elders of the past whose signatures of past environmental and social conditions provide a lens to understand historical processes and acknowledge their significance beyond mere scientific specimens. Thus, ancient remains deserve respect *ìwà ìtẹríba* (respect). As researchers advance into the data collection phase, they must embrace *ìwà ìrẹ̀lẹ̀* (humility) and establish connections with community members as equals. Having the financial resources for research does not confer superiority over those being studied. It is vital to approach community members respectfully, actively listen to their concerns, and be willing to learn from them. Additionally, humility is a quality that fosters effective collaboration between researchers and community members (Schreiber and Tomm‐Bonde 2015). This attribute helps build trust within the community and plays a pivotal role in the success of the research.

After data have been successfully collected and are being interpreted, researchers must ensure that the representation of the people is accurate and truthfully represented (*òtítọ́, ọ̀rọ̀ sísọ*) and that there is a consciousness of the people being understudied (Figure 1). This is vital, knowing the risks involved for diverse populations when using genetic data (Choudhury et al. 2020; Fu et al. 2014; Wolf and Akey 2018). Data interpretation from the Yoruba community demands a conscientious and culturally sensitive approach. Herein lies the necessity for the *Ọmọlúàbí́* principle to serve as a useful framework for interpreting genetic data based on Indigenous cultural dynamics. This approach effectively bridges the divide between raw data and its contextual relevance within the Yoruba culture. Genetic data interpretation should be inextricably intertwined with the cultural milieu of the population under investigation. Yoruba Indigenous hermeneutics embodies a comprehensive comprehension of the Yoruba worldview, values, traditions, and historical context—thereby enabling a nuanced understanding of the data in a manner that respects and resonates with the Yoruba people's cultural heritage. When researchers engage with data sourced from the Yoruba community, they encounter a multifaceted challenge: the need to avoid divorcing the data from the cultural tapestry in which it is embedded. To accomplish this, researchers must ensure that research findings are scientifically accurate and socially meaningful, fostering trust and collaboration between researchers and the Yoruba community. This cultural grounding avoids misrepresentation or oversimplification of genetic findings that could perpetuate harmful stereotypes or contribute to cultural erasure.

Data sovereignty and sharing are the primary aspects of research that have consistently led to ethical concerns. As with other research phases, how the data are treated is critical (Figure 1). At the core of this issue lies *ìwà ìtẹríba* (respect), which ensures that communities maintain sovereignty over their data, narratives, and cultural representations. Researchers must adopt sensitive data‐sharing practices that acknowledge communities' inherent right to control how their information is used and disseminated. *Òtítọ́* (truth) reinforces the importance of transparency and honesty in the research process. Researchers must accurately represent the data's origins, context, and implications, avoiding any misrepresentation or distortion that might serve external agendas. Truthfulness also requires acknowledging the contributions of Indigenous knowledge systems and the involvement of community members in the research process. By aligning with these principles, researchers uphold the integrity of their work, fostering trust and collaboration with the Yoruba community. Furthermore, the dissemination of research findings must account for the ethical implications of publication (Figure 1), requiring the application of *ìronú* (critical thinking) and *ọ̀rọ̀ sísọ̀* (culturally grounded speech), especially understanding that how data are presented can impact the community. Careful consideration is necessary to prevent misrepresentation or harm, and also to understand what data may be publicly shared. The values of *mímọ̀ rírí àwùjọ* (giving back to the community) and *ajọṣe* (communalism) further emphasize the importance of reciprocity and collaboration. By prioritizing benefits such as shared intellectual property, co‐authorship opportunities for local researchers, or contributions to community development, research avoids perpetuating extractive practices and instead promotes equity and cultural preservation.

To accomplish this, the *Ọmọlúàbí* ideology is an invaluable guide, advocating for a holistic approach from initial study design through data interpretation and dissemination. The principle ensures that researchers engage in practices that are respectful, reciprocal, and attuned to the lived realities of the Yoruba community while acknowledging the interconnectedness of genetic data and cultural realities. By integrating the moral and ethical tenets of *Ọmọlúàbí* into the interpretation process, researchers can navigate the complex terrain of Yoruba culture with sensitivity and authenticity. Moreover, this approach aligns research with broader ethical values and creates a global model for ethical, culturally informed genetics research. Finally, researchers must note that understanding the culture‐specific common morality of the people will not occur by reading books or published literature about the community, but through community involvement, extensive communication and discussion, and by learning from participant observation.

## 5 Conclusions

Incorporating the *Ọmọlúàbí́* ideology is an example of an Indigenous hermeneutic useful for genetics research. It necessitates a departure from conventional paradigms that often prioritize a detached, objective stance. Instead, the integration of culturally specific concepts of common morality, such as Ọmọlúàbí́ among the Yoruba people of Southwestern Nigeria, calls for deeper researcher engagement to recognize the interplay between culture, identity, and data—especially when such data are as sensitive as genetic information. In this context, data are not a sterile entity but a living artifact intricately interwoven with Indigenous cultures' lives, identities, values, norms, and history.

We do not propose Ọmọlúàbí́ as a wholesale replacement for existing ethical frameworks such as Principlism, FAIR, or CARE principles. Rather, it represents a culturally specific moral framework that operationalizes these high‐level principles by providing detailed guidance on how ethical obligations are interpreted, enacted, and evaluated within Yoruba contexts. In this way, Ọmọlúàbí́ functions not as an alternative paradigm but as an example of a culturally grounded mechanism that completes and localizes broader, global ethical commitments, making them meaningful and actionable for local communities.

Integrating the *Ọmọlúàbí́* ideal within genetics research practices with the Yoruba people can aid in transcending the confines of data interpretation and extend its influence into the rich realm of cultural narrative. By embracing the *Ọmọlúàbí́* way of conducting genetics research with the Yoruba people, as an example (others include *Tikanga Māori* of the Māori, the Indigenous Polynesian people of New Zealand) (Hudson et al. 2010), *Inuit oaujimajatuqangit* of the Inuit, inhabiting North America's Arctic and subarctic regions (Nickels and Knotsch 2012; Tagalik 2011), Ubuntu of Southern Africans (Seehawer 2018), *suban* of the Akan of Ghana (Gyekye 1995), researchers can effectively navigate the culturally laden landscape of research ethics and participatory research paradigms. Such an approach facilitates a more equitable and respectful engagement with research participants, positioning them as co‐creators and collaborators in the research process and the findings and minimizes harm to communities. Consequently, it enhances the quality and ethical integrity of research endeavors. It promotes cultural sensitivity and reciprocity in research conducted within culturally diverse and richly textured contexts like that of the Yoruba people. Overall, genetics researchers must develop a deep‐seated relationship with the communities under study, exposing them to the people's common morality and promoting beneficial partnerships.

## Author Contributions

**Iyunoluwa J. Ademola‐Popoola:** conceptualization, writing – original draft, funding acquisition, writing – review and editing. **Laura S. Weyrich:** conceptualization, writing – review and editing, writing – original draft.

## Funding

This work was supported by National Science Foundation (DGE1255832).

## Conflicts of Interest

The authors declare no conflicts of interest.

## Data Availability

Data sharing not applicable to this article as no datasets were generated or analysed during the current study.

## Endnotes

^1^
Indigenous in this context of the Yoruba is referred to as groups who are natives and or who share collective ancestral ties and are owners of land (Ogundiran 2020).

^2^
Ọmọlúàbí‐pronounced as aw‐maw‐loo‐ah‐bee.
